# Uropathogenic *Escherichia coli* in Recurrent Urinary Tract Infection: Intracellular Persistence, Immune Evasion, Antimicrobial Resistance, and Non-Antibiotic Interventions

**DOI:** 10.3390/antibiotics15090847

**Published:** 2026-08-31

**Authors:** A. L. Sreelekshmi, Abhijith Pulimoottil Jaykumar, Pradeesh Babu, Aravind Madhavan, Bipin G. Nair, Geetha B. Kumar

**Affiliations:** Amrita School of Biotechnology, Amrita Vishwa Vidyapeetham, Clappana P.O., Kollam 690525, Kerala, India; am.ls.r4bio23423@am.students.amrita.edu (A.L.S.); am.ls.r4bio24440@am.students.amrita.edu (A.P.J.); pradeeshbabu@am.amrita.edu (P.B.); aravindmi@am.amrita.edu (A.M.); bipin@am.amrita.edu (B.G.N.)

**Keywords:** Uropathogenic *E. coli*, immune evasion, pathogenesis, recurrent UTIs, treatment, epidemiology, antimicrobial resistance

## Abstract

Uropathogenic *Escherichia coli* (UPEC) remains the principal driver of urinary tract infections (UTIs), utilizing sophisticated immunoevasive strategies that consistently outmaneuver host defenses and conventional clinical management. This review synthesizes current evidence highlighting the development of recurrent UTIs (rUTIs). Particular emphasis is given to the transition of UPEC into intracellular bacterial communities (IBCs) and quiescent intracellular reservoirs (QIRs)—survival phenomena primarily characterized across specialized mammalian and cellular models. By highlighting the translational evidence gaps surrounding these intracellular sanctuaries, and the challenge of directly extrapolating these model-derived dynamics to human recurrent UTIs (rUTIs), alongside the role of extra-urinary niches, we provide a comprehensive view of the mechanisms driving recurrence. A critical assessment of the UPEC virulence arsenal, involving molecular mechanisms that regulate fimbrial phase variation resulting in evading host immune surveillance, to specialized adhesins, like FimH, which tightly bind uroplakin receptors, triggering host actin rearrangements via rho-GTPase signaling and facilitating bacterial internalization, is also addressed. Additionally, we integrate the roles of cytotoxic necrotizing factor-1 (CNF1) and hemolysin A (HlyA) in promoting cytosolic escape, host cell survival, and long-term persistence. Finally, this review presents a multidisciplinary framework evaluating next-generation non-antibiotic strategies, including bacteriophage therapy, vaccines, phytotherapy, probiotics, and estrogen therapy. By critically assessing their efficacy and clinical maturity across a spectrum of preclinical models and human trials, we evaluate the potential to navigate translational limitations to effectively disrupt the infection cycle.

## 1. Introduction

Urinary tract infections (UTIs) are amongst the most prevalent, frequently recurring illnesses in the world, posing a considerable risk to human health. According to Global Burden of Disease estimates, the annual incidence of UTIs in general increased from approximately 2.7 billion cases in 1990 to 4.5 billion cases in 2019, representing a 66.45% rise worldwide, with the highest incidence reported in Africa reme caution in high-risk groups. This includes immunocompromised patients, those on anti-rejection or chemotherapy medications, individuals with structural heart disease or a history of endocarditis, and patients with acute intestinal [1,2,3,4].

Women are known to have the highest occurrence of UTIs, with over 26% of women experiencing a recurrence of UTIs within 6 months of being cured [5,6,7]. This heightened female susceptibility is driven by anatomical factors, a short urethra and close proximity of the perianal region to the vulvar vestibule, which facilitate the migration and colonization of common intestinal uropathogens, like *Escherichia coli* [8]. Additionally, behavioral and physiological variables, like sexual activity, thickness of vaginal epithelium and mucus layer, vaginal pH, and estrogen, as well as glycogen levels and diversity of the gut microbiome, such as the presence of *Lactobacillus* species, also influence the likelihood of UTI development in women [9].

UTIs arise from infection by a broad spectrum of bacterial pathogens, such as *E. coli*. *Klebsiella pneumoniae*, *Proteus mirabilis*, *Pseudomonas aeruginosa*, *Staphylococcus saprophyticus*, *Staphylococcus aureus,* and *Enterococcus* species, as well as fungal pathogens, like *Candida* spp. Furthermore, a substantial fraction of cases are polymicrobial UTIs, characterized by the presence of multiple uropathogens in midstream urine at titers > 100,000 CFU/mL [10]. Despite this diversity, *E. coli* is the most prevalent, contributing to about 80–95% of the reported cases [11,12]. Specifically, Uropathogenic *Escherichia coli* (UPEC) is the predominant etiological agent of UTIs, accounting for more than 75% of all cases [13,14].

UTIs are normally treated using a plethora of antibiotics such as fluoroquinolones, beta-lactams, sulfonamides, fosfomycin, and nitrofurantoin. However, therapeutic failure and recurrent UTIs are rising rapidly. This clinical bottleneck is driven by a dual challenge: the exponential global spread of antimicrobial resistance (AMR) and the intrinsic ability of UPEC to subvert host immunity by establishing protected intracellular reservoirs. Standard oral antibiotics frequently fail to eradicate UPEC sequestered inside urothelial cells, leaving quiescent bacterial populations that trigger recurrent episodes.

Consequently, to break the cycle of recurrent UTIs, it is imperative to consider alternative strategies such as natural products, hormone therapy, probiotics, phage therapy, and vaccines that disrupt UPEC pathogenesis.

To ensure mechanistic clarity throughout this review, it is critical to precisely define the distinct pathobiological terms driving UPEC pathogenesis. Clinically, a recurrent UTI is defined as two or more symptomatic UTIs within a six-month period, or three or more within one year. This serves as an umbrella term encompassing two distinct etiologies: relapses and reinfections [15]. A relapse is the re-emergence of the original pathogen after achieving sterile urine, typically driven by survival within protected bladder niches [16]. Conversely, a reinfection represents a newly established infection originating from an extra-urinary reservoir-anatomical sites, such as the gastrointestinal tract or vagina, where pathogens safely reside between acute episodes [16]. In these niches, uropathogens frequently exist in a state of asymptomatic colonization. Clinically, this manifests as asymptomatic bacteriuria, which is strictly defined by the presence of at least 10^5^ colony-forming units per milliliter (CFU/mL) of a uropathogen in voided urine in the absolute absence of clinical signs [17]. This is distinguished from a symptomatic infection (such as acute uncomplicated cystitis), which is diagnosed only when bacterial presence is actively accompanied by host tissue damage and overt clinical symptoms [18].

Resistance refers specifically to heritable, genetic antimicrobial resistance (AMR) that allows bacteria to actively proliferate in the presence of antibiotics [19]. In contrast, bacterial persistence is an adaptive, non-heritable phenotypic state where a dormant subpopulation, known as persister cells, successfully evades both immune surveillance and pharmacological eradication. Antibiotic tolerance is a survival strategy where the main bacterial population transiently survives under normally lethal antibiotic concentrations without any change in the minimum inhibitory concentration (MIC). Unlike resistance, which permits active bacterial growth during exposure, tolerance is simply characterized by a slower antibiotic kill rate [20,21,22].

While the existing literature extensively discusses UPEC-driven UTI epidemiology and the AMR crisis, a critical knowledge gap remains. Contemporary reviews frequently maintain a strictly bladder-centric focus and extrapolate murine model findings to human pathology without highlighting translational limits, particularly regarding intracellular bacterial communities (IBCs). Furthermore, previous papers often provide descriptive inventories of alternative therapeutics rather than rigorously assessing their clinical viability.

This review bridges these gaps by offering an integrated, critical framework. Unlike prior reviews, we explicitly differentiate between in vitro, murine, and human clinical data to prevent overgeneralizing rUTI mechanisms, synthesizing these dynamics into a novel visual model. We expand the traditional scope by examining the extra-urinary niches (gastrointestinal and vaginal reservoirs) that drive reinfection. Finally, rather than merely cataloguing countermeasures, we critically analyse the clinical efficacy, technological maturity, and specific translational limitations of next-generation interventions, including bacteriophages, estrogen, phytotherapy, probiotics, and vaccines. By synthesizing high-resolution pathogenic mechanisms with a rigorous translational critique, this review provides a mechanistically grounded roadmap currently absent from the literature.

## 2. Methodology for Selecting the Scientific Literature

Articles used to prepare the current review were searched using the PubMed, Google Scholar, and ClinicalTrials.gov databases, supplemented by citation-network mapping with ResearchRabbitai. In that respect, only English-language peer-reviewed articles were included.

The search terms and Boolean operators were combinations of “urinary tract infections” OR “UTIs” OR “uropathogenic *Escherichia coli*” OR “UPEC” AND “intracellular bacterial communities”, “quiescent intracellular reservoirs”, “pathogenesis”, “antimicrobial resistance”, or “novel treatments”.

Search results were primarily focused on the literature published within the last 20 years (2006–2026), though seminal older studies were retained for foundational biological context. The selection encompassed in vitro studies, animal models, clinical trials, case reports, and established clinical guidelines. Non-peer-reviewed preprints and conference abstracts were excluded. Discrepancies across studies were evaluated and synthesized by considering experimental designs, strain variations, and clinical contexts.

## 3. From Reservoir to Urothelial Invasion: The Pathogenic Trajectory of UPEC

Before UPEC can establish a recurrent infection, it must first emerge from its primary ecological niche, navigate host defenses, and successfully invade the urinary tract. This progression relies on a sequential combination of epidemiological transmission, evolutionary selection, and sophisticated molecular subversion.

### 3.1. Transmission and Pathogenic Selection

The most common immediate source for the infecting UPEC is the endogenous fecal (and, in female patients, vaginal) microbiota of the host [23]. However, in humans, these internal microbial reservoirs are frequently seeded by external environmental sources, including contaminated retail meat products and localized transmission within households and sexual networks [24,25,26,27,28,29,30,31]. Moving from this highly diverse gut reservoir to the sterile urinary tract serves as an evolutionary bottleneck. Ultimately, colonization of UPEC is driven by two complementary concepts. The prevalence hypothesis attributes UTIs to the most numerically abundant *E. coli* in the gut, while the pathogenicity theory posits that strains are evolutionarily selected for enhanced virulence. Ultimately, both mechanisms operate synergistically to drive infection [14]. 

### 3.2. Clinical Phenotypes of UTIs

Once UPEC successfully navigates these pathogenic mechanisms to establish an infection, the resulting clinical presentation can vary widely. *E. coli*-mediated UTIs manifest differently depending on patient demographics and bacterial entry points. Therefore, understanding their specific classifications is crucial for both diagnosis and targeted treatment. Generally, these are categorized by anatomical site (lower UTIs like cystitis vs. upper UTIs like pyelonephritis), clinical complexity (uncomplicated in healthy individuals vs. complicated in immunocompromised), and acquisition setting (community vs. hospital-acquired) [32,33,34]. While catheter-associated hospital infections involve a broader array of resistant pathogens (*K. pneumoniae*, *P. aeruginosa*), UPEC remains the dominant etiological agent across almost all phenotypes, responsible for 75% of uncomplicated and 65% of complicated UTIs [12,35].

### 3.3. The Immunological Landscape and Host Subversion

Regardless of whether a UTI presents as an uncomplicated localized infection or a highly complicated, upper-tract disease, the underlying success of the pathogen relies on a shared set of sophisticated survival strategies. To establish these diverse clinical phenotypes across different anatomical sites, UPEC must first navigate a hostile immunological landscape and actively subvert the host’s innate defense mechanisms with the help of an arsenal of virulence factors.

The pathogenesis typically initiates with surface factors, most notably type 1 and P fimbriae, which enable the pathogen to anchor to specific urothelial or kidney epithelial receptors, withstand the mechanical shear force of voiding, and trigger host cell invasion. Following successful attachment, UPEC deploys secreted toxins, such as α-hemolysin (HlyA) and cytotoxic necrotizing factor 1 (CNF1), to inflict targeted cellular damage, manipulate host apoptotic pathways, and liberate essential intracellular nutrients.

Furthermore, they utilize capsular polysaccharides and flagella to evade innate immune clearance. Rather than acting in isolation, these factors operate synergistically to drive the distinct phases of UPEC pathogenesis. A comprehensive breakdown of these virulence determinants, their specific molecular targets, and their mechanistic roles in clinical outcomes is synthesized in Table 1. Likewise, the transition from initial encounter to rUTI depends on the bacteria’s virulence factors and transcriptional adaptation to subvert host defenses and establish protected intracellular reservoirs as observed in murine models.

#### 3.3.1. The Interplay of Fimbrial Phase Variation, Host Immunity, and Pathogenic Outcomes

To survive shifting host pressures during initial colonization, UPEC relies on *fimbrial phase variation*. This stochastic “Phase-ON/Phase-OFF” switching is driven by the site-specific inversion of the *fimS* DNA element, governed by tyrosine recombinases (*fimB/fimE*) and nucleoid-associated proteins [61]. Ultimately, the state of these fimbrial structures governs how the host immune system responds to the invasion.

#### 3.3.2. Acute Urothelial Defense: The Tug of War in the Bladder

**Figure 1:**
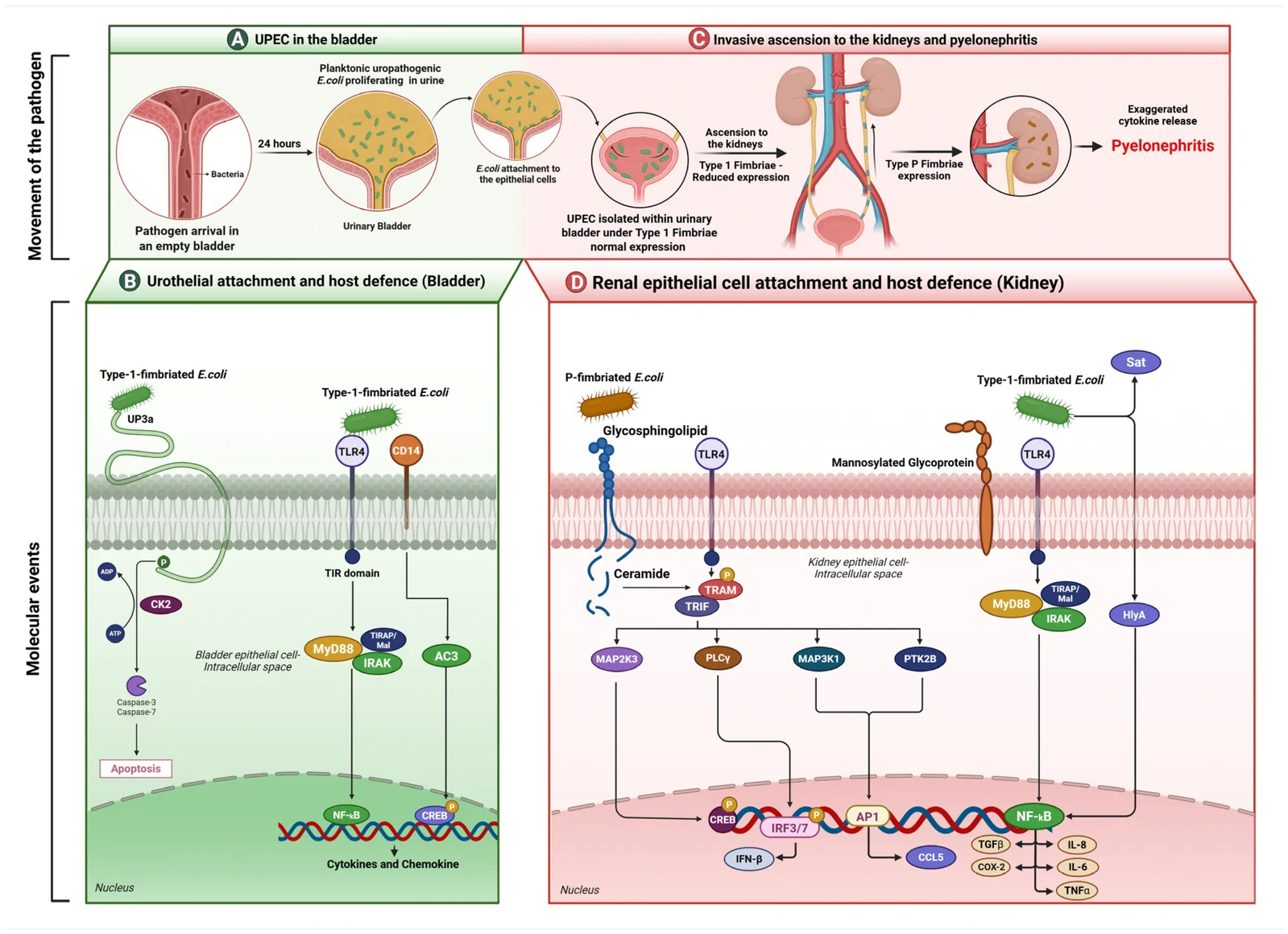
The progression of UPEC infection from bladder colonization to pyelonephritis. (the upper panel shows the overall movement of the UPEC, and the lower panels depict the molecular events). Panel (**A**) shows the arrival of UPEC in the bladder followed by its proliferation in the urine and attachment to the bladder epithelial cells. Panel (**B**) depicts the molecular events following attachment of UPEC to uroplakin A receptors, leading to apoptosis, or TLR4 and CD14 receptors, leading to cytokine production and pro-inflammatory response. Panel (**C**) shows the ascension of UPEC from the bladder to the kidney, leading to pyelonephritis. Panel (**D**) depicts the molecular events following the attachment of UPEC to the TLR4 receptors in the renal epithelial cells and the cascade of events leading to the pro-inflammatory response.

Under “phase-on” conditions, type 1 fimbriae mediate the initial attachment to mannose moieties of uroplakin receptors on urothelial umbrella cells facing the bladder lumen within 4 to 24 h of infection [62,63]. However, the host recognizes this attachment as a danger signal, simultaneously triggering two parallel intracellular pathways, the canonical TLR-4/MyD88 pathway and the alternate AC-3/CREB pathway, to eliminate the threat, as shown in Figure 1B.

**The canonical TLR-4/MyD88 pathway:** The host monitors the extracellular space via Toll-like receptor 4 (TLR-4). Assisted by the co-receptor CD14, TLR-4 recognizes bacterial lipopolysaccharide (LPS). LPS engagement activates the intracellular TIR (Toll/interleukin receptor-1) domain of TLR-4, recruiting the adaptor proteins MyD88 and TIRAP/MAL to form an active signaling complex. MyD88 recruits IL-1 receptor-associated kinase (IRAK), which downstream activates tumor necrosis factor receptor-associated factor 6 (TRAF-6). TRAF-6 mediates the phosphorylation and degradation of IκB, releasing the transcription factor NF-κB. Free NF-κB rapidly translocates to the nucleus to initiate the transcription of pro-inflammatory cytokines and chemokines [64].

**The alternate AC-3/CREB pathway:** In addition to the canonical MyD88 pathway, TLR-4 utilizes an alternate signaling arm via adenylate cyclase-3 (AC-3) activation. AC-3 converts intracellular ATP to cAMP, activating protein kinase A (PKA), which subsequently phosphorylates the cAMP response element-binding protein (CREB). Phosphorylated CREB enters the nucleus, binds directly to cAMP response elements in the DNA, and works in tandem with NF-κB [64]. Together, they drive a robust upsurge in cytokine and chemokine production that recruits polymorphonuclear leukocytes (PMNs/neutrophils) to the bladder [64].

However, UPEC can counteract these defenses by exploiting the rapid apoptosis and exfoliation process. The bacteria may actually bypass the superficial layer to gain access to deeper, underlying tissue, where the immune response is less effective [65].

#### 3.3.3. Invasive Ascension: The Breach into the Renal Parenchyma

As depicted in Figure 1D, upon ascending to the kidneys, UPEC shifts its fimbrial expression. Binding of the PapG class II adhesin to glycosphingolipid receptors (GbO4) on kidney epithelial cells induces the localized release of lipid ceramide [66]. Ceramide release stimulates TRAM (TRIF-related adaptor molecule) phosphorylation and activates the mitogen-activated protein (MAP) kinase pathway, which drives the nuclear translocation of alternative transcription factors, specifically IRF-3, AP-1, and CREB, resulting in a massive, localized production of pro-inflammatory cytokines, including IL-6 and IL-8. Clinical pyelonephritis occurs when the subsequent renal cytokine signaling becomes exaggerated or dysfunctional [67]. This cytokine storm often leads to host tissue damage, which is further worsened by secreted bacterial toxins, such as hemolysin A (HlyA) and sat toxin. HlyA boosts the production of inflammatory cytokines, like IL-6 and IL-8, and lyses renal cells to release iron for bacterial use [68,69]. Sat toxin is a vacuolating cytotoxin that directly impairs glomeruli and is cytopathic to the surrounding renal epithelium [68,69]. In severe cases, the bacteria penetrate the endothelial cells of the proximal tubules and enter the bloodstream, converting a localized tissue infection into life-threatening bacteremia [70]. Notably, while type 1 fimbriated *E. coli* strains can trigger standard MyD88-dependent NF-κB signaling if present in the renal niche, the direct contribution of P-fimbriated *E. coli* to this standard MyD88 pathway is remarkably weak [66,67].

## 4. Recurrent UTI, Intracellular Reservoirs, and Immune Evasion: The Trojan Horse Strategy

One of the most clinically challenging aspects of UTIs is their high rate of recurrence. Following an initial UTI, 20–30% of female patients endure a recurrence, with a smaller fraction suffering from chronic, nearly continuous infections [71,72].

To definitively differentiate between a dormant bladder reservoir and an external reservoir, multi-omics studies can be employed. High-resolution whole-genome sequencing (WGS) reveals that in humans, persisting UPEC lineages undergo subtle, niche-specific evolution mediated by mobile genetic elements, depending on whether they are adapting to the gastrointestinal or urinary tract. As there is bidirectionally informed communication between the gut, urinary tract, and genital tract, studies must collect paired samples from the urine, feces, and vaginal tracts of patients with UTIs to accurately map the cross-habitat migration of the pathogen. *E. coli* residing in the gastrointestinal region can be identified from the stool, whereas urine contains *E. coli* from the urinary tract. Longitudinal tracking of patients’ microbiome over extended periods also aids in observing temporal dynamics of uropathogens [73].

When evaluated through this high-resolution framework, recurrent UTIs can be mapped to four distinct pathways:

1. **Intra-vesical relapse:** Emergence from persistent mucosal niches, specifically intracellular bacterial communities (IBCs) or quiescent intracellular reservoirs (QIRs) within the urothelium, following antibiotic cessation [74].
2. **Gastrointestinal reinfection:** Continuous auto-inoculation and upstream migration from the gut reservoir, where genetically identical UPEC strains systematically reseed the urinary tract via fecal–perineal–urethral transmission [75]. This dynamic exposes a critical vulnerability in empirical therapeutics, like nitrofurantoin; while it achieves high bactericidal concentrations in urine to clear bladder-resident strains, its poor intestinal tissue penetration leaves the gastrointestinal pathogen bunker completely undisturbed [76]. Post-treatment recurrence thus frequently reflects an unaddressed gut niche rather than true intra-tissue bladder failure.
3. **Persistence-associated recurrence:** Driven by a metabolic strategy where a subpopulation of “persister cells” exhibits temporary, non-heritable antibiotic tolerance by reverting to an inactive, non-replicating state during active therapy, allowing them to survive lethal drug exposures independent of macro-structural protection [77].
4. **Vaginal epithelial relapse:** Evidenced as a critical, underrecognized extra-urinary reservoir where UPEC invades the vaginal epithelium to escape extracellular stressors and aminoglycoside clearance [78]. Vaginal intracellular communities (VICs) serve as a protective staging ground that promotes long-term pathogen longevity and facilitates subsequent ascension into the urinary tract, making the vaginal niche a vital variable in the success of future non-antibiotic therapies.

### 4.1. The Evidence Hierarchy of Intra-Vesical Invasion and QIRs

**Figure 2:**
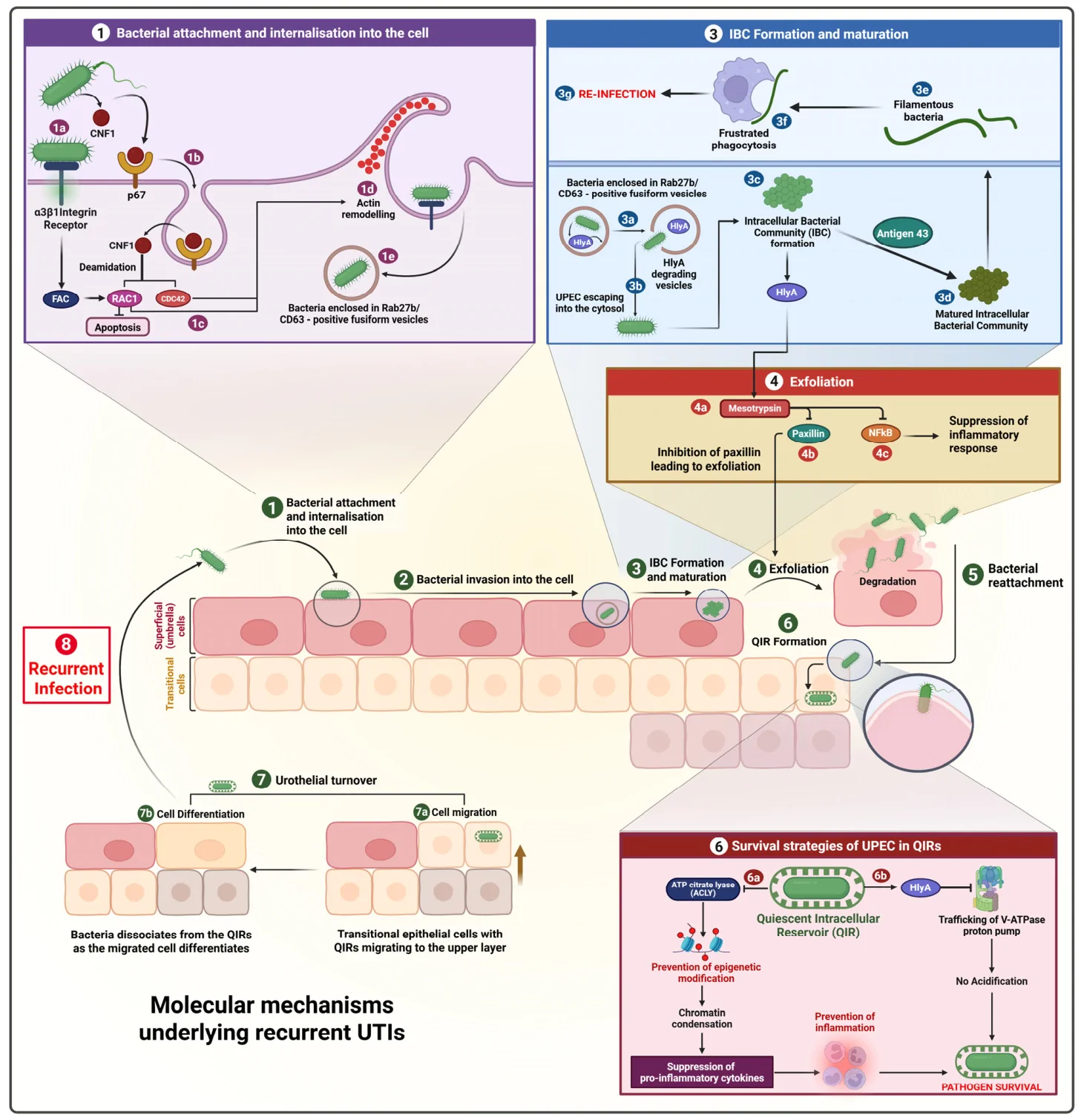
Synthesized model of recurrent UTIs: (**1**) Bacterial attachment and internalization into the cell, (**1a**) attachment of UPEC to α_2_β_3_-integrin receptor, (**1b**) receptor mediated endocytosis of CNF-1, (**1c**) molecular basis of actin remodeling, (**1d**) active toxin mediated entry via invagination of cell membrane, (**1e**) passive mechanical entry via internalization of UPEC into the Rab27b/CD63-positive endosomes; (**2**) UPEC invasion into the cell; (**3**) IBC formation and maturation: (**3a**) bacterial production of HlyA and pore formation in the vesicle, (**3b**) escape of pathogens from the vesicles, (**3c**) formation of IBCs, (**3d**) antigen 43-mediated maturation of IBCs, (**3e**) formation of extracellular filamentous bacteria, (**3f**) immune evasion, (**3g**) re-infection; (**4**) exfoliation: (**4a**) HlyA-mediated activation of mesotrypsin, (**4b**) mesotrypsin mediated inhibition of paxillin leading to exfoliation, (**4c**) mesotrypsin mediated inhibition of NF-κB and suppression of inflammatory responses; (**5**) re-attachment of UPEC to transitional cells; (**6**) Survival strategies of UPEC in QIRs: (**6a**) prevention of inflammation via inhibition of ACLY, (**6b**) inhibition of V-ATPase and prevention of lysosome acidification; (**7**) urothelial turnover: (**7a**) migration of transitional cells, (**7b**) differentiation of the migrated cells and QIR release; (**8**) recurrent infection.

While UPEC exploits various anatomical reservoirs to seed reinfection, its localized intracellular lifecycle within the bladder epithelium, transitioning from initial receptor engagement to IBC formation and ultimately QIR establishment, represents the most extensively studied and widely recognized mechanism of UPEC recurrence. However, evaluating this mechanism requires strictly separating findings derived from animal models from direct clinical evidence in humans. The intracellular lifestyle of UPEC within the bladder is a highly orchestrated cycle of invasion, replication, and persistence. Because no single experimental model captures the entirety of this process in humans, we have synthesized in vitro, murine, and clinical data into a composite visual model of UPEC bladder recurrence (Figure 2).

#### 4.1.1. Active and Passive Entry Mechanics

Infection establishment begins with UPEC binding α_3_β_2_-integrin receptors on bladder epithelial cells, stimulating focal adhesion kinase (FAK) and activating Rho family GTPases, like RAC1, which drives host actin rearrangement and initiates internalization, as depicted in Figure 2 [79]. UPEC subverts host clearance through two distinct entry repertoires: active toxin-mediated entry and passive mechanical entry.

**Active toxin-mediated entry:** This process is driven by the RfaH-regulated activity of CNF1 and HlyA, which forces host cell engulfment via Rho GTPase activation while simultaneously blocking apoptosis to keep the host cell alive (Figure 2, Panel 1) [56,80,81,82,83]. CNF1 binds explicitly to the host’s p67 laminin receptor and the Lutheran (Lu) blood group molecule, entering the cytoplasm via clathrin-independent endocytosis [81]. Inside the cytosol, CNF1 deamidates the Gln63 residue of Rho GTPases, constitutively locking Rho, RAC1, and CDC42 into an active state [56,83]. This triggers massive actin rearrangement, generating a zipper-like mechanism that forces the host cell membrane to engulf the adherent bacteria [56]. Crucially, by sustaining downstream pro-survival signaling through RAC1, CNF1 actively prevents host cell apoptosis, overriding the default exfoliation defense mechanism to maintain a long-lived intracellular niche for the pathogen [80]. Mechanistically, this pathway is firmly established in human cell culture models, while its overarching importance to *E. coli* pathogenesis is corroborated by murine infection models [84].

**Passive mechanical entry:** Parallel to active entry, UPEC can seamlessly exploit the host bladder’s natural mechanical voiding cycles. In a healthy bladder, Rab27b/CD63-positive fusiform vesicles fuse with the apical membrane to facilitate expansion. Evidence from both cultured human bladder epithelial cells 5637 and murine infection models indicates that UPEC may utilize fusiform vesicles as an intracellular niche, with cAMP-regulated exocytosis potentially contributing to bacterial persistence and recurrence. The natural drop in cyclic AMP (cAMP) during bladder contraction triggers the ruffling of the apical membrane, passively carrying UPEC into the cell within these specialized vesicles (Figure 2, Panel 1e) [85].

#### 4.1.2. Divergent Fates: IBC Formation and Nutritional Immunity

Once sequestered within the vesicles, the pathogen’s fate is determined by its interaction with host sensors. While host TLR-4 senses UPEC and induces cAMP to promote bacterial exocytosis, UPEC subverts this clearance pathway by disrupting the endosomal vesicle membrane to escape into the host cytoplasm, where it subsequently aggregates to form an intracellular bacterial community (IBC) (Figure 2, Panel 3) [12,86]. In both murine and human bladder epithelial cell models, HlyA has been shown to regulate this circuitous path by promoting escape from RAB27b+ fusiform vesicles into the host cytosol [87]. In vitro studies in human bladder epithelial cells further show that HlyA also inserts into epithelial cells and macrophages, activating mesotrypsin and host serine proteases to degrade proteins, like paxillin and NF-κB, leading to epithelial exfoliation and the suppression of inflammatory responses (Figure 2, Panel 4) [57,58].

To fuel rapid replication within the IBC, UPEC must bypass nutritional immunity, where the host secretes lipocalin-2 (Lcn-2) to sequester iron [88,89]. Mouse model studies demonstrate that UPEC counters this through the Ferric. Uptake Regulator (FUR) rheostat, upregulating salmochelin, which evades Lcn-2, and aerobactin [90,91].

Furthermore, murine models show that as the IBC matures, bacteria are embedded in a polysaccharide matrix and utilize antigen 43 for dense packing. By 16 h post-infection, the acute intracellular lifecycle culminates in host cell lysis, triggering the synchronized fluxing of bacteria into the bladder lumen. During this dispersal phase, a subpopulation of UPEC fails to septate and differentiates into elongated filamentous forms on the bladder surface, which serve to subvert neutrophil engulfment via frustrated phagocytosis [92,93]. These filamentous bacteria subsequently undergo nutrient-independent septation to release normal rod-shaped daughter cells. This newly liberated population drives subsequent, tandem rounds of reinfection by invading healthy superficial umbrella cells to establish second-generation IBCs, which exhibit distinct adaptations to the intracellular niche, including significantly slower growth kinetics and extended doubling times (Figure 2, Panel 3) [94].

**Clinical relevance and evidence gaps**

While the theory of IBC formation offers a compelling explanation for the persistence of *E. coli* within the bladder wall, its real role in human rUTI remains a subject of active debate. The formation of IBCs and QIRs is well-established in murine models and has been supported by microscopic evidence in human exfoliated cells in urine [65,95,96,97,98]. However, recent evidence suggests these findings may not universally translate across different species. For instance, a porcine model of UTI failed to demonstrate stable IBC formation, with bacteria failing to survive mecillinam treatment, which challenges the notion of IBCs as an impenetrable sanctuary [99]. Consequently, while the intracellular niche remains an important area of research, further clinical evidence is required to confirm that these reservoirs are the primary drivers of recurrence in the human host rather than transient phenomena.

#### 4.1.3. Quiescent Intracellular Reservoirs (QIRs): Mechanism of Persistence

Following IBC dispersal, a subset of UPEC is spread to deeper epithelial cells to establish quiescent intracellular reservoirs (QIRs), as evidenced from mouse models [94]. The existence of these latent stages, where the bacteria reside within non-acidified LAMP-1-positive endosomes surrounded by F-actin, is primarily evidenced by murine models [57,100].

Mechanistic insights from human bladder epithelial cells and a murine C57BL/6J model reveal that HlyA impairs lysosomal acidification by disrupting the microtubule-dependent trafficking of V-ATPase, thereby enhancing intracellular UPEC survival [87,101]. By inhibiting the microtubule-dependent transport system required for the V-ATPase to reach the vesicle, HlyA ensures the lysosomal environment remains at a neutral, non-lethal pH, thereby facilitating long-term survival. The therapeutic potential of targeting this host–pathogen interface was recently demonstrated in mouse models, where the pharmacological disruption of microtubules successfully restored the host’s ability to clear these reservoirs, significantly reducing the intracellular bacterial burden (Figure 2, Panel 6) [87].

Additionally, both cell line and murine bladder data demonstrate that UPEC further secures its survival by inhibiting the host enzyme ATP citrate lyase (ACLY), a key provider of the acetyl-CoA required for histone acetylation. By depleting this essential enzyme for epigenetic modification, the pathogen effectively locks the host’s chromatin in a condensed state, suppressing the transcription of pro-inflammatory cytokine genes and preventing an inflammatory clearance response [102]. Under this combined shield of neutralized vesicular pH and a silenced immune landscape, UPEC enters a state of metabolic dormancy [103]. These dormant bacteria are resistant to antibiotics and remain undetected for months until urothelial turnover, triggered by the migration and differentiation of basal cells, releases them to reinitiate the infection cycle [96,104].

#### 4.1.4. Advanced In Vitro Platforms and Intratissue Niches of Persistence

While definitive human validation of IBCs and QIRs remains challenging, researchers are increasingly deploying sophisticated human in vitro bladder platforms to bridge these evidence gaps—fundamentally reshaping our understanding of UPEC pathogenesis, structural tissue niche utilization, and recurrence biology. In a recent study, a human bladder-chip model that co-cultures superficial umbrella cells and microvascular endothelial cells under continuous physiological urine and media flow was developed. Real-time time-lapse microscopy from this system revealed that while vascular neutrophil recruitment triggers intense local swarming and neutrophil extracellular trap (NET) formation, these acute host defenses are entirely bypassed by the intracellular bacterial community (IBC). It was also seen that the bacteria inside IBCs were difficult to clear using antibiotics [105].

Parallel investigations using human bladder organoids to simulate a multi-layered, stratified urothelium demonstrated that UPEC rapidly penetrates deep beyond the superficial barrier to form an architecturally isolated subpopulation of solitary, flagellated, rod-shaped bacteria. Protected from both antibiotic exposure and neutrophil killing, these deep-seated solitary bacteria diverge morphologically from classical biofilm-like IBCs. Notably, their immediate appearance in deeper tissues challenges the long-held paradigm that sequential cycles of superficial cell exfoliation are strict prerequisites for deep invasion, highlighting a highly accelerated path to persistence [106].

Complementary insights from three-dimensional human urothelial microtissue (3D-UHU) and multi-layered transwell architectures have clarified that intracellular invasion is a widely conserved survival strategy shared by both pathogenic UPEC and commensal *E. coli* strains. While the type 1 fimbrial adhesin FimH remains mandatory for the orchestration of highly structured IBCs, it is dispensable for initial cell entry; furthermore, commensals actively employ alternative intracellular phenotypes like filamentation and the hijacking of exfoliating host cells. However, host cells respond discriminately, aggressively expelling invasive commensals while pathogenic strains trigger severe barrier degradation, exfoliation, and profound pro-inflammatory cytokine cascades [107].

To integrate the chemical microenvironment with these structural and dynamic mechanical inputs, a human mini-bladder model exposing a stratified urothelium to real urine compositions under active voiding cycles demonstrated that prolonged exposure to highly concentrated, high-solute urine significantly reduces tissue resilience, disrupting tight junctions and dysregulating immune responses to promote UPEC colonization. Crucially, within this high-solute mini-bladder niche, antibiotic pressure actively drives UPEC to transition into stable, cell wall-deficient (CWD) L-forms within the deep urothelial layers, providing a highly recalcitrant structural reservoir that underpins recurrent UTIs [108]. Together, these modern, human-relevant microphysiological platforms bridging structural biology, urine chemistry, and fluid dynamics bridge the gap between animal models and actual clinical recurrence.

## 5. The Central Crisis: Antimicrobial Resistance in UPEC

AMR is a critical contributor to the elevated death rates and healthcare costs coupled with UTIs. Globally, an estimated 4.95 million deaths were associated with bacterial AMR in 2019, with *E. coli* being the single pathogen causing the highest number of these deaths across all types of infections [109]. In 2019, *Escherichia coli* was the leading pathogen associated with AMR-related mortality in UTIs, accounting for approximately 40% of such fatalities, with an estimated 26,520 deaths directly attributable to AMR [110]. The clinical and public health consequences of escalating AMR extend far beyond mortality. The prevalence of AMR among UPEC isolates against formerly dependable first-line oral antibiotics has reached alarming levels worldwide, rendering standard empirical regimens increasingly ineffective. Consequently, patients infected with multidrug-resistant strains frequently experience delayed clinical resolution, recurrent emergency department visits, prolonged hospitalizations, and an increased reliance on expensive, broad-spectrum intravenous therapies. For instance, infections caused by extended-spectrum β-lactamase (ESBL)-producing *E. coli* represent an independent risk factor for early clinical failure, with the overall economic cost of managing these resistant episodes nearly doubling compared to susceptible infections [111]. Together, these diagnostic and therapeutic challenges impose a profound financial strain on healthcare systems while severely diminishing patient quality of life.

The prevalence of AMR among UPEC isolates to formerly dependable first-line antibiotics has reached threatening levels worldwide, making many common empirical treatments ineffective.

To explore the profound impact of AMR on urinary tract infection therapy, we examine the four critical pillars of UTI management, trimethoprim–sulfamethoxazole, beta-lactams, fluoroquinolones, nitrofurantoin, and fosfomycin, each representing a distinct molecular battlefield where UPEC has evolved sophisticated survival mechanisms.

The survival of UPEC against clinical intervention begins with resistance to antifolates, such as trimethoprim–sulfamethoxazole (TMP-SMX). These agents act as competitive inhibitors of enzymes in the folic acid synthesis pathway (Fol pathway), where SMX inhibits dihydropteroate synthase (DHPS), and TMP inhibits dihydrofolate reductase (DHFR) [112]. In the USA, current UPEC susceptibility levels of 70–80% often breach the 20% resistance ceiling, disqualifying these antibiotics as dependable first-line empirical therapies [113]. The main mechanism of resistance to TMP-SMX is the acquisition of plasmid-encoded genes, particularly from the *dfrA* and *sul* families, which encode altered versions of DHFR and DHPS with reduced affinity for the drugs [114]. These genes are frequently located on integrons and transposons, enabling their horizontal transfer among UPEC strains [115,116]. Additionally, chromosomal mutations in the native *dhfr* and *dhps* genes can modify the three-dimensional structure of these enzymes, decreasing drug binding affinity while maintaining their functional activity [115].

This pattern of evasion extends to beta-lactams, which inhibit bacterial cell wall formation by binding to essential enzymes known as penicillin-binding proteins (PBPs) [117]. Resistance in UPEC arises from the production of beta-lactamase enzymes, target site mutations, and the action of efflux pumps [1,118]. The production of beta-lactamases, specifically the CTX-M, TEM, and SHV families of ESBLs, as well as AmpC enzymes, represents the most significant mechanism, as these enzymes cleave the integral beta-lactam ring [118,119,120]. Alarming resistance to carbapenems is driven by a diverse array of carbapenemases. In UPEC, this includes metallo β-lactamases, like the New Delhi metallo β-lactamase (NDM), Verona integron-encoded metallo β-lactamase (VIM), and imipenemase (IMP), alongside serine carbapenemases, such as *Klebsiella pneumoniae* carbapenemase (KPC) and OXA-48-like enzymes. The acquisition of these enzymes is particularly concerning within the virulent B2 phylogroup, where up to 100% of isolates can exhibit multidrug resistance [121,122] Additionally, mutations in the *mrdA* gene reduce target affinity, while the overexpression of efflux pumps, such as AcrAB-TolC, actively expels carbapenems from the cell [1,2]. To counteract these defenses, therapeutic strategies utilize combinations, like ceftazidime/avibactam, to inhibit ESBL activity [123].

Fluoroquinolones, including ciprofloxacin and levofloxacin, have historically served as first-line empirical therapies for UTIs due to their broad-spectrum efficacy, favorable pharmacokinetic profiles, and excellent tissue penetration, yielding high urinary concentrations [124]. Mirroring an escalation in global resistance trends, recent localized epidemiological data from North India highlight a severe baseline shift, with fluoroquinolone resistance rates reaching an alarming 70.2% among uropathogens—a trajectory predominantly driven by resistant strains of *Escherichia coli* (76.1%) [124]. This resistance primarily drives clinical treatment failure through target site modifications, mediated by chromosomal mutations within the *gyrA* and *parC* quinolone resistance-determining regions (QRDRs), as well as horizontal acquisition of plasmid-mediated *qnr* mechanisms. Ultimately, this resistant phenotype is heavily selected for and disseminated by clinical risk factors such as prior antibiotic exposure, instrumentation via catheterization, and underlying host comorbidities [124,125,126].

In contrast to the rising resistance in other classes, nitrofurantoin maintains relatively low resistance rates, recorded at 7.6% globally [127]. Its efficacy is derived from its reduction into bioactive metabolites by bacterial oxygen-insensitive nitroreductases, encoded by *nfsA* and *nfsB* [128,129]. These metabolites bind to ribosomes to inhibit translation and disrupt the citric acid cycle [130]. Resistance typically requires chromosomal mutations in *nfsA*, *nfsB*, or the *ribE* gene [131]. However, plasmid-mediated mechanisms have emerged, including the OqxAB efflux pump and mutated CTX-M-14 beta-lactamases that have evolved to hydrolyse nitrofurantoin [132,133].

Finally, fosfomycin remains an effective option by disrupting peptidoglycan synthesis through the competitive inhibition of the MurA enzyme at the Cys-115 residue [134]. Resistance to this agent is primarily conferred through a mutation of the Cys-115 residue, which prevents drug binding. Acquisition of plasmid-borne *fos* genes (e.g., *fosA3*, *fosA8*, *fosL1*) can also chemically inactivate the molecule, while mutations in cellular transport systems prevent the absorption of the antibiotic [135,136]. These collective mechanisms demonstrate the diverse evolutionary strategies UPEC employs to circumvent modern antimicrobial therapy, justifying their role as the primary focus of this discussion.

## 6. Alternative Strategies to Treat UTIs

**Figure 3:**
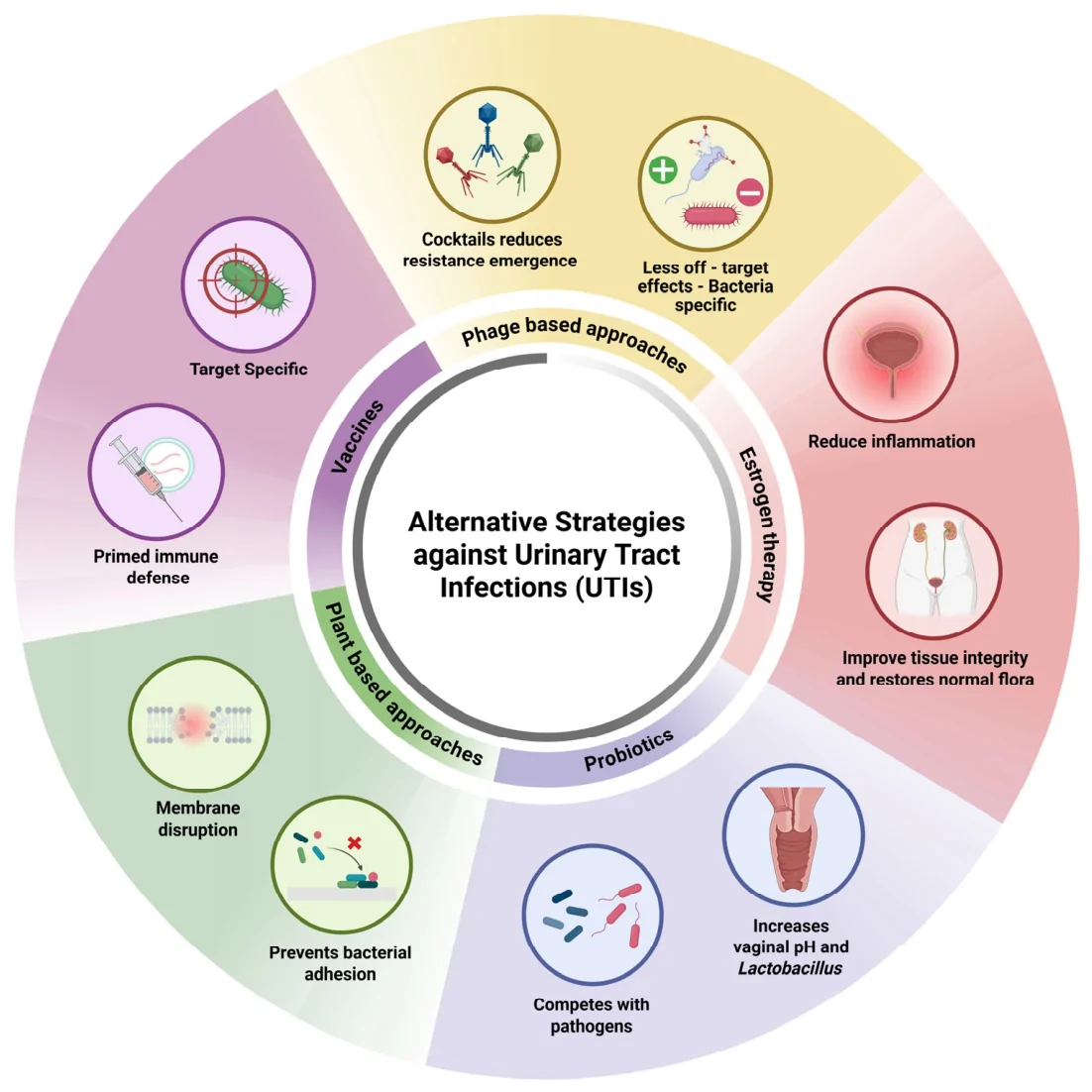
Overview of alternative therapeutic strategies for UTIs, illustrating the mechanisms of vaccines, phage therapy, estrogen treatment, probiotics, and plant-based approaches in targeting pathogens and restoring host physiological balance.

As there is an exponential increase in UPEC resistance to commonly used antibiotics to treat UTIs, there is an urgent need to explore alternative strategies to combat this problem. These emerging interventions can be critically categorized into two distinct mechanistic pathways: pathogen-directed therapies (such as phages and phytotherapy) that neutralize bacterial virulence, and host-directed modalities (including probiotics, estrogen, and vaccines) that restore the tissue microenvironment and prime immune defenses, as conceptualized in Figure 3. A comprehensive synthesis of these alternative strategies, critically evaluating their mechanisms, preclinical progress, and current clinical maturity, is detailed across Table 2a–d, revealing a transition from experimental pilot studies to promising synergistic applications.

### 6.1. Phages

Phage therapy, utilizing viruses that specifically target bacterial pathogens, represents a compelling therapeutic option for UTIs, particularly those caused by multidrug-resistant (MDR) UPEC. Bacteriophages are naturally abundant, often outnumbering bacteria by a 10:1 ratio, and can be rapidly isolated from environmental sources [137]. Unlike broad-spectrum antibiotics, phages offer the capacity for self-replication with minimal dysbiosis to the host microbiome [138]. Additionally, as highlighted in Table 2b, their ability to produce depolymerases allows them to disrupt biofilms and decrease bacterial adherence, making them a potent tool against the characteristic intracellular survival of MDR-UPEC [139,140].

**Table 2:** Alternative strategies for the management of UTIs and their clinical maturity.

Category	Strategy	Experimental Evidence (In Vitro)	Preclinical Studies (In Vivo)	Clinical Studies	References
(**a**) Vaginal estrogen therapy with stronger guidelines and clinical support in postmenopausal recurrent UTI					
Hormonal therapy	Estrogen	Estrogen treatment of human urothelial cell lines enhances antimicrobial peptide expression and cell–cell contact proteins, preventing UPEC adherence and invasion into deeper epithelial layers.	Ovariectomized mice show an increase in recurrent UTI-seeding reservoir.		[141,142,143,144]
(**b**) Phage therapy and vaccines; promising but uneven in clinical maturity					
Phage therapy		Bacteriophage cocktails provide a potent defense against MDR UPEC by significantly reducing biofilm adherence and viability while simultaneously driving evolutionary trade-offs that increase bacterial susceptibility to antibiotic treatment.	Clears UPEC from urine and tissues (mouse/rat models); reduces urogenital colonization; synergistic with antibiotics to prevent regrowth.	Successful eradication of recurrent ESBL *E. coli* in case studies; RCTs demonstrate safety, tolerability, and rapid reduction in *E. coli*, though some trials show comparable success to placebo.	[139,145,146,147,148,149,150,151,152,153,154,155,156,157]
Prophylaxis	Vaccine	FimH vaccines (adjuvanted with TLR-4) successfully induce and qualify highly specific, functional FimH-binding antibodies in validated assays.	mRNA nanoparticle (FimH-Ferritin) and recombinant multi-peptide (PapG/FimH) constructs elicit robust systemic, mucosal, and T-cell immune responses in rodent models.	Sublingual MV140 drastically reduces UTI episodes and healthcare costs; ExPEC10V exhibits functional opsonophagocytic killing; StroVac reduces UTI frequency but requires further placebo-controlled validation.	[158,159,160,161,162,163,164]
(**c**) Phytotherapy strategies evaluated via preclinical mechanisms and small clinical trials					
Plant products	Cranberry	Oligosaccharides and phenolics reduce UPEC biofilm and adherence; upregulate tight junction proteins to protect epithelial barriers.	Juice, hydrophilic fractions, and M-3-Gal reduce *E. coli* colonization in mouse bladders; reduces bacterial adherence in cats.	Significantly reduces clinical UTI incidence, recurrence, and antibiotic use in women and children.	[165,166,167,168,169,170,171,172,173,174,175,176]
	Bearberry	Tinctures demonstrate antibacterial activity against uropathogens, like *S. aureus* and *E. coli.*	Not available	Decreases antibiotic consumption in uncomplicated cystitis; symptom relief is comparable to placebo; some trials noted slightly higher pyelonephritis risk versus antibiotics.	[177,178,179,180,181]
	Pomegranate	Peel extracts inhibit growth, biofilm formation, adherence, and β-lactamase production of *E. coli* and *S. aureus*; synergistic with antibiotics.	Not available	Pilot studies show combination therapy (with D-Mannose and probiotics) significantly improves acute cystitis symptoms and quality of life.	[182,183,184,185]
	Green Tea	Catechins, nanoemulsions, and ethanolic extracts inhibit UPEC growth and biofilm formation.	Not available	Adjunctive therapy alongside standard antibiotics significantly decreases cystitis symptoms and improves urinalysis.	[186,187,188,189]
	Oregano	Essential oils and fractionated extracts inhibit growth and motility (swimming/swarming) of *E. coli*, *P. aeruginosa*, and *P. vulgaris*; bactericidal against MDR strains.	Not available	Not available.	[190,191,192,193,194]
	Roselle	Calyx extracts inhibit UPEC biofilm capacity and exhibit concentration-dependent bacteriostatic/antibacterial activity.	Oral administration suppresses LPS-induced NF-κB activation and renal inflammation in mice.	Herbal combinations containing hibiscus reduce UTI incidence, relieve symptoms, prevent recurrence, and serve as an antibiotic-sparing approach.	[195,196,197,198,199,200,201]
	Thyme	Thymol inhibits biofilm formation, downregulates *fimH* expression, and enhances antibiotic sensitivity (cefotaxime, ciprofloxacin).	Aqueous extracts demonstrate prominent antimicrobial efficacy in a rat pyelonephritis model.	Not available.	[202,203,204]
(**d**) Probiotics with strain specificities and variable evidence					
Microbiome	Probiotics	*L. plantarum* biofilms inhibit *E. coli* adhesion to silicone; *L. crispatus* provides cytoprotection; *L. rhamnosus* GR-1 potentiates immune responses (NF-κB).	*L. crispatus* limits bladder UPEC infection in mice by triggering a host type I interferon response to clear intracellular bacteria.	Reduces UTI recurrence and delays the time to first symptomatic UTI; does not increase antibiotic resistance; enhances efficacy when combined with antibiotics.	[205,206,207,208,209,210,211,212]

To mitigate the rapid evolution of bacterial resistance, clinical application typically involves phage cocktails, a combinational strategy that significantly reduces the probability of resistance development [213]. Modern evolutionary models also indicate that when UPEC develops phage resistance, it frequently incurs a fitness cost, rendering the pathogen more susceptible to the host immune system and existing antibiotics [138]. This has led to the rise of Phage–Antibiotic Synergy (PAS), where sub-inhibitory antibiotic concentrations facilitate accelerated phage progeny release and optimized bacterial lysis [214]. Even temperate phages, traditionally avoided due to their potential for lysogeny, can be leveraged in PAS; sublethal antibiotic doses can successfully shift phage dynamics toward the lytic cycle to eradicate MDR pathogens [215].

#### 6.1.1. Translational Hurdles: Biological and Pharmacological Limitations

Despite this potential, several biological constraints limit the seamless transition of phage therapy from the laboratory to the clinic [216]. Clinically, the rapid lysis of bacteria can trigger a dangerous release of endotoxins, potentially leading to systemic septicemia [217]. A primary hurdle is the extreme difficulty in getting regulatory approval. Furthermore, achieving efficient systemic phage penetration into hidden intracellular niches, such as deep intra-vesical urothelial reservoirs, remains an unresolved challenge. Conversely, recent evidence shows successful decolonization of UPEC from the vaginal and urogenital epithelium, effectively minimizing the pathogen load in these vital upstream staging grounds [149].

Another important consideration in phage therapy is the distinction between lytic and temperate phages. Lytic phages infect bacterial cells, rapidly replicate, and ultimately lyse the host bacterium to release progeny phages, making them the preferred candidates for therapeutic applications. In contrast, temperate phages can enter a lysogenic cycle, during which the phage genome integrates into the bacterial chromosome as a prophage and replicates along with the host cell without immediate bacterial killing. This lysogenic state may facilitate horizontal gene transfer and potentially disseminate virulence factors, toxin genes, or AMR determinants among bacterial populations [218,219].

Prior to phage therapy, phage genomes should be effectively sequenced, assembled, annotated, and screened for antibiotic resistance using tools like Resfinder or Resistance gene identifier and virulence genes using VirulenceFinder [220,221,222,223].Temperate phages possess risks associated with the ability to integrate harmful genes, such as AMR genes, into the bacterial hosts. Also, they prevent superinfection by other phages in the infected host by giving superinfection immunity [224]. Consequently, temperate phages are generally considered less suitable for therapeutic use [218,219].

Pharmacologically, phages face rapid degradation by the host’s metabolic and immune systems. The mammalian circulatory system effectively clears phage particles, while the gastrointestinal barrier often dilutes therapeutic concentrations, complicating the establishment of consistent pharmacokinetic data [225,226]. Moreover, there exists profound disparity between in vitro replication dynamics and in vivo realities, where phage kinetics are governed by a complex, multi-variable matrix. Unlike static chemical antibiotics, in vivo phage viability and therapeutic efficacy are fundamentally dependent on intrinsic viral parameters, such as the adsorption rate, latency period, burst size, and initial dosage, juxtaposed against host factors. These host constraints include regional anatomophysiology, environmental conditions at the infection site, systemic tissue distribution, and the clearance rate of viral particles by the host immune system. Furthermore, the capacity of phages to replicate in situ is continuously challenged by the localized emergence of bacterial phage resistance, rendering predictable mathematical modeling of phage pharmacokinetics and pharmacodynamics exceptionally difficult [227].

Phage therapy is a heterogeneous field with different product standards and clinical trial quality. Product standards vary drastically, creating a wide gap between unstandardized, non-GMP (Good Manufacturing Practice) personalized formulations used in compassionate care and highly pure, GMP-grade fixed cocktails required for scalable regulatory approval [228]. Also, the quality of available clinical evidence remains highly uneven [229].

Furthermore, an evaluation of clinical maturity (Table 2b) highlights a dichotomy between preclinical efficacy and human trials. While case reports show successful eradication of MDR *E. coli*, robust data from randomized controlled trials (RCTs) present highly uneven results, frequently confounded by the concomitant use of standard antibiotics, making it difficult to definitively isolate the independent therapeutic impact of the phages. Although there are case reports on the safety and efficacy of phage therapy, robust data from randomized controlled trials (RCTs) are limited and frequently confounded by the concomitant use of standard antibiotics, making it difficult to definitively isolate the independent therapeutic impact of the phages [230].

#### 6.1.2. The Global Landscape of Phage Therapy: Clinical Maturity and Regulatory Stratification

The global landscape of phage therapy is highly stratified, reflecting significant differences in regional regulatory frameworks and historical acceptance. In Eastern Europe, most notably Georgia and Russia, phage therapy is a mature, standard-of-care intervention utilizing licensed, broad-spectrum medicinal cocktails that bypass the rigid clinical trial prerequisites mandated by Western agencies [231]. Conversely, Western regulatory bodies, such as the US FDA and the European EMA, classify phages as complex biological medicinal products, restricting their use to rigorous randomized controlled trials or highly regulated compassionate use pathways, although Belgium has pioneered a flexible magistral compounding approach for personalized preparations [232].

Bridging this divide, Asia represents a dynamic transitional model, particularly in China and India. In China, commercially developed phage formulations with defined compositions are classified as innovative biologics and must undergo Investigational New Drug (IND) approval through the Center for Drug Evaluation (CDE) under the National Medical Products Administration (NMPA) before they can be used in industry-sponsored clinical trials. Conversely, personalized therapies follow the Investigator-initiated Trials (IIT) pathway, where successful results allow medical institutions to apply for restrictive medical technology status via Provincial Health Commissions [233]. Similarly, India is formalizing national guidelines to integrate its historical phage infrastructure into modern frameworks for clinical practice and compassionate use.

### 6.2. Phytotherapy: The Botanical Arsenal Against Uropathogens

As outlined in Table 2c, plant-based alternatives, including herbs, berries, leaves, and roots, are increasingly recognized as strategic options for UTI management due to their diverse anti-infective mechanisms [234]. Extracts isolated from these medicinal plants exhibit a clinical triad of antimicrobial, anti-inflammatory, and antioxidant activities [235]. A primary advantage of these chemically complex compounds is their ability to inhibit microbial growth through molecular pathways distinct from conventional antibiotics, offering significant therapeutic value in treating resistant strains [236].

Hydrophobic essential oils derived from aromatic plants and specific secondary metabolites, such as the proanthocyanidins (PACs) abundant in cranberries (*Vaccinium subg. oxycoccus*), represent premier options for UTI prevention. PACs specifically target P and type 1 fimbriae to prevent pathogen adhesion while simultaneously inhibiting biofilm formation and exhibiting synergistic effects with D-mannose, which competitively blocks the FimH adhesin [165,168,175]. Additional alternatives include bearberry, which contains arbutin that metabolizes into antimicrobial hydroquinone in alkaline urine, and pomegranate extracts, which demonstrate robust antibacterial activity against multidrug-resistant (MDR) *E. coli*.

Other botanicals with recognized anti-infective mechanisms include lingonberry (*Vaccinium vitis-idaea*), roselle (*Hibiscus sabdariffa*), green tea (*Camellia sinensis*), and essential oils from oregano (*Origanum vulgare*), thyme (*Thymus vulgaris*), and goldenrod (*Solidago virgaurea*) [234]. Furthermore, the integration of plant extract–antibiotic synergistic combinations contributes to a reduction in necessary antibiotic dosages and combating MDR infections [237]. The overarching potential of these plants is anchored in biochemical synergy; the emergence of multi-target mechanisms and compounds capable of suppressing resistance allows for enhanced bioavailability and reduced systemic toxicity [238]. Unlike synthetic drugs, these complex natural products demonstrate a lower propensity for the development of bacterial resistance since they rarely depend on a single molecular target [239,240].

#### Translational Limitations of Phytotherapy

Despite these advantages, the transition of phytotherapy from traditional use to standardized clinical practice faces profound hurdles. The primary disadvantage lies in the lack of standardization and inconsistent bioavailability. Unlike synthetic antibiotics, herbal extracts contain a complex mixture of phenolics, flavonoids, and tannins whose concentrations vary drastically based on the plant’s origin, harvest time, and extraction method [241]. As reflected in Table 2c, there is a severe translational gap in phytotherapy. While these extracts demonstrate substantial in vitro biological activity, most interventions lack continuous in vivo validation, relying heavily on small pilot studies rather than standardized RCTs. Among these, cranberry products are the most extensively investigated and clinically relevant for UTI prevention. However, their therapeutic efficacy is not uniform; clinical trial outcomes vary extensively across different product formulations and distinct patient populations.

Also, the bioavailability of these compounds is often unpredictable. Many active phytochemicals require specific pharmacokinetic profiles to ensure they reach the urinary tract in therapeutic concentrations without being degraded by systemic metabolism [242]. Sustainability also remains central to these interventions; variability in plant material and harvesting pressure can directly affect compound quality and cost [243].

The safety and toxicity of phytochemicals necessitate a critical assessment. As these compounds may be used over extended periods or at high doses to achieve antimicrobial efficacy, they require thorough toxicity screening and pharmacokinetic studies [244]. Additionally, the scalability of production is often curtailed by laborious extraction procedures and variable yields from natural sources [245]. While biotechnology, including metabolic engineering and controlled cultivation, is increasingly used to improve consistency, the clinical demand for these therapies remains largely unmet due to these technical barriers.

Furthermore, a major translational limitation of phytotherapy is its reliance on single-pathogen models, which neglect the ecological complexity of the urinary microbiome. Natural urological infections often present as polymicrobial communities rather than isolated monocultures. Bacterial species within polymicrobial UTIs form intricate interaction networks that govern community stability and promote collective antibiotic tolerance. Within these networks, cooperative microbial behaviors can shield otherwise vulnerable uropathogens from clearance. Consequently, while botanical extracts or essential oils may successfully eliminate isolated pathogens like *E. coli* in vitro, their efficacy can be compromised or rendered unpredictable by the collective resilience of a multi-species ecosystem [246].

### 6.3. Gut Microbiota, Probiotics, and UTI Prevention

The gut microbiota plays a critical role in the development of UTIs, acting as a primary reservoir for uropathogens. A recent study demonstrated that the resistance profile and clonal identity of gut-colonizing *E. coli*, alongside the age of the carrier, can predict the risk of contracting a UTI within an 18-month period [247]. This study, involving women with no prior antibiotic exposure or recent hospitalizations, found that fecal *E. coli* carriers were significantly more prone to UTIs than non-carriers, strongly suggesting that *E. coli* serves as the primary pathogen in these infections [247].

To counter this, probiotics can be utilized to manage the gut environment. While the majority of dietary fibers reach the colon intact, probiotics assist in their fermentation, producing antimicrobials like bacteriocins and short-chain fatty acids that reduce the growth of harmful microbes. They also boost host immunity via TLR activation. Beyond dietary fermented options, like Kefir, Kombucha, Yogurt, Miso, Tempeh, Sauerkraut, Kimchi, and Natto [237], recurrent UTIs can also be prevented through fecal microbiota transplantation to restore ecological balance [248].

#### 6.3.1. Lactobacillus and UTI Prevention

As evaluated in Table 2d, probiotic efficacy demonstrates high translational consistency, where in vitro mechanisms of competitive exclusion seamlessly translate to in vivo immune modulation and successful human clinical trials. Probiotic effects are highly strain-specific, which means that beneficial properties demonstrated by one specific bacterium cannot be generalized to all strains within the same species. *Lactobacillus* species are highly effective as probiotics for UTI prevention. The bacteriostatic effect of these probiotics is primarily achieved through direct competition with uropathogens for limited nutrients and a finite number of attachment sites. For instance, epithelial adherence can be achieved by surface proteins and fatty acids, as observed for *L. rhamnosus* Pen, or by proteinaceous surface layer components, as seen in *L. plantarum* 91 [249,250].

Direct bactericidal activity is also strain-dependent; for example, *Lactobacillus johnsonii* NCC933 and *Lactobacillus gasseri* KS120.1 strains have been shown to directly kill *Escherichia coli* CFT073, a urinary tract pathogen [251] (Atassi & Servin, 2010). Furthermore, the urogenital probiotic strains *L. rhamnosus* GR-1 and *L. reuteri* RC-14 combat UPEC by secreting lactic acid and hydrogen peroxide. These by-products heavily inhibit UPEC growth, induce membrane stress (upregulating porins *ompA* and *ompX*), and downregulate *fimA* and *papA* fimbrial genes to disrupt UPEC host tissue attachment [252]. Additionally, *E. coli* biofilms challenged with the secreted products of *L. rhamnosus* GR-1 exhibit a marked decrease in cell density and increased cell death [253].

Clinically, oral administration of *L. rhamnosus* GR-1 and *L. reuteri* RC-14 has proven to reduce pathogens in the vagina, help reduce recurrence, and mitigate AMR [254]. For targeted delivery, intravaginal suppositories containing *Lactobacillus crispatus* CTV05, *L. rhamnosus* GR-1, and *L. reuteri* RC-14 are particularly effective against uropathogens, showing the greatest efficacy for UTI prophylaxis while being well-tolerated with no major side effects [255].

#### 6.3.2. Clinical Challenges and Safety Considerations of Probiotics

Despite these benefits, the clinical application of probiotics involves several challenges. While generally considered safe and used in fermented foods for centuries, probiotics are primarily regulated as dietary supplements rather than drugs [256]. Common side effects include mild gastrointestinal distress, such as bloating, gas, diarrhea, and nausea, which typically resolve as the microbiome readjusts [257,258]. More critically, as live microorganisms, probiotics carry an inherent risk of causing systemic infections that require antibiotic treatment. This risk is higher if strains are genetically modified or possess antibiotic selection markers that could be transferred to other bacteria [256]. Consequently, probiotic supplementation must be used with extreme caution in high-risk groups. This includes immunocompromised patients, those on anti-rejection or chemotherapy medications, individuals with structural heart disease or a history of endocarditis, and patients with acute intestinal disease or perforations [259].

### 6.4. Estrogen Therapy

The increased risk of UTIs in women can be largely attributed to estrogen deficiency occurring after menopause, which often leads to vaginal prolapse, incomplete bladder emptying, and urinary incontinence [260]. Similar to probiotics, estrogen therapy (Table 2a) presents a seamless investigational pipeline, where in vitro observations of enhanced antimicrobial peptide expression perfectly align with in vivo clearance of recurrent UTI reservoirs. Vaginal estrogen has been proven to reduce vaginal pH, restore the protective *Lactobacillus* population, and improve the tissue integrity of the genitourinary region. While urothelial exfoliation is a natural defense mechanism used to shed pathogens, excessive exfoliation can inadvertently result in recurrent infections by facilitating the formation of QIRs and IBCs, as previously discussed. To overcome this, vaginal estrogen therapy has been shown to increase levels of IL-6 and reduce urinary inflammation [260].

Currently, the most frequently prescribed formulation is a vaginal cream administered via a plastic intravaginal applicator [261]. By restoring the natural urogenital barriers, vaginal estrogen offers a strategic way to minimize both therapeutic and prophylactic antibiotic use, ultimately helping to avoid the development of resistant bacteria [143].

#### Clinical Risks and Adverse Effects of Estrogen Therapy

While estrogen therapy can offer clear benefits for urinary tract health, it is not risk-free. The side effects of estrogen therapy are dependent on the dosage and route of administration and whether the route has local or systemic effects. Generally, high doses can lead to fluid retention, vaginal bleeding, and even mental status changes [262]. A mouse study with high estrogen doses (4.2 μg/day) has shown to negatively impact heart and kidney functioning, whereas a lower dosage of 0.02 μg/day partially restored uterine weight and did not adversely affect the kidney but, in fact, tended to offer cardio-renal protection in ovariectomized mice [263]. Patients must be evaluated on an individual basis to determine if the UTI prevention benefits outweigh these systemic risks. Even when used alone, common side effects include breast tenderness, bloating, nausea, headaches, leg cramping, and vaginal bleeding [262]. These factors underscore the necessity of a personalized approach when utilizing estrogen to manage UTIs.

### 6.5. Vaccines

Vaccination represents a transforming solution to manage UTIs, moving away from reactive antibiotic use toward a strategy of primed immune defense. The core objective of vaccines is to stimulate the host’s immune system, specifically targeting UPEC to recognize and neutralize pathogens before they can colonize the bladder mucosa or ascend to the kidneys. Current vaccine strategies are broadly classified based on their composition: whole-cell, polysaccharide conjugate, and subunit formulations. OM-89 (Uro-Vaxom) is an oral immunomodulator composed of fractionated bacterial components rather than intact cells; it functions primarily by activating the gut-associated lymphoid tissue (GALT) to stimulate innate immune pathways and mucosal secretory IgA [264]. Whole-cell vaccines, including Urovac and Uromune, are the most clinically established. Their primary advantage is the presentation of a complete antigenic repertoire, which triggers a broad immune response against multiple bacterial strains [265,266]. However, their limitation was evident in early live-attenuated versions, like CP923 and NU14 ΔwaaL, which suffered from rapid clearance and failed to protect renal tissues because surface polysaccharides sterically shielded underlying proteins [267,268].

To address this, polysaccharide conjugate vaccines (e.g., ExPEC10V) were developed to turn short-lived immune responses into long-term memory by linking bacterial sugars to carrier proteins [158,162]. Meanwhile, subunit vaccines focus on specific virulence factors, components that the bacteria use, such as the FimH adhesin. While highly precise, these face the limitation of pathogen heterogeneity, as seen in the discontinuation of FimH trials due to insufficient coverage of diverse bacterial strains [269,270]. The future effectiveness of these vaccines likely depends on their integration with alternative, non-antibiotic therapies to create a comprehensive defense system.

Evidence suggests that vaccines work best when combined with agents that physically block bacterial binding, such as D-mannose or mannosides [271,272]. By pairing vaccination with rescue interventions like bacteriophage therapy or utilizing probiotics to maintain a healthy urogenital microbiome, clinicians can provide a robust alternative to antibiotics [273,274]. This transition toward a vaccine-centered, synergistic model is essential for reducing the selective pressure of antibiotics and ensuring long-term success in UTI prevention [275]. Table 3 illustrates that investigational study types directly dictate regulatory success. Traditional whole-cell formulations (e.g., Uromune, Urovac) bypass complex molecular modeling for broad empirical clinical studies, achieving high clinical maturity and commercial licensing. Conversely, precision-engineered approaches (e.g., ExPEC10V), despite molecular superiority in preclinical models, currently remain restricted to early-phase trials due to pathogen heterogeneity.

#### Vaccination: Limitations

Vaccine efficacy is primarily limited by primary failure, where individuals—often due to genetics or immunodeficiency—fail to develop an initial immune response, and secondary failure, where protective immunity fades over time without boosters. External factors like strain variation, improper storage, and technical administration errors further compromise clinical success. Additionally, potential side effects ranging from localized inflammation to hypersensitivity reactions highlight gray areas in long-term safety that necessitate rigorous monitoring [285].

## 7. Limitations of the Review

While this review provides a comprehensive synthesis of Uropathogenic *E. coli* pathogenesis and emerging non-antibiotic interventions, several inherent boundaries must be acknowledged. First, a significant portion of the molecular data detailing the intracellular lifestyle of UPEC, including IBC and QIR dynamics, relies heavily on murine and specialized in vitro models, which still face translational constraints and lack uniform histopathological validation across large human clinical cohorts. Second, the evaluation of alternative therapeutic strategies, such as phage therapy, probiotics, and phytotherapy, is limited by the highly heterogeneous and uneven nature of the available literature; many cited clinical successes are derived from small pilot studies or case reports often confounded by the concomitant use of standard antibiotics. Finally, because this manuscript intentionally focused on UPEC as the primary driver of recurrence, it does not fully address the distinct pathogenic networks of other prominent uropathogens or the unique management guidelines required for complicated, catheter-associated infections.

## 8. Conclusions

The global burden of UTIs is mainly caused by UPEC, whose clinical trajectory is controlled by a formidable array of virulence factors. UPEC achieves critical colonization of different anatomical sites by regulating surface appendages, such as type 1 fimbriae, while its nutrient acquisition and tissue invasion are facilitated by secretion of toxins and siderophores. The persistence of UPEC is further exacerbated by its ability to survive in an intracellular state and establish intracellular bacterial communities (IBCs) for rapid expansion or quiescent intracellular reservoirs (QIRs) for long-term dormancy, effectively shielding the pathogen from immune clearance and antibiotic therapy. UPEC continues to develop robust resistance mechanisms against frontline antibiotics, such as TMP-SMX, fluoroquinolones, fosfomycin, nitrofurantoin, and β-lactams, creating a serious threat to public health, leading to increased treatment failures and mortality. A potential solution to this UPEC-centered crisis is exploring novel strategies such as bacteriophage therapy, probiotics, and hormone therapy. rUTIs could also be tackled by developing new intracellular drug delivery systems to target hidden niches of UPEC.

## Figures and Tables

**Table 1:** Virulence factors of UPEC and mode of action.

Virulence Factors		Mode of Action	Reference
Surface factors	Type 1 fimbriae	Binds to mannose receptors on the urothelium, aids in biofilm and IBC formation	[36,37,38,39,40,41]
	Type 2 or P fimbriae	Attaches to globotriaosylceramide (GbO4) receptors on epithelial cells	[42,43,44]
	Dr adhesin	Binds to kidney epithelial cells and Bowman’s capsule	[45]
	S fimbriae	Associates with sialic acid residues on host bladder epithelial cells, as well as on the endothelium of the bladder and kidneys	[46]
	F1C fimbriae	Attaches to 4-Gal-β and Gal-NAc-β-1 moieties on the host urinary mucosal membrane, including the ureters, nephrons, urothelial cells, glomeruli, and the endothelial cells of the bladder and kidneys, thereby promoting biofilm formation	[41,47,48]
	Flagella	Motility, invasion of renal collecting duct in pyelonephritis, aids in bacterial movement from bladder to the kidneys	[49,50]
	Capsule	Protection from the immune system, phagocytosis, and complement-mediated removal; capsules like K1 and K5 mimic host tissue, hence preventing humoral immune responses	[51]
	LPS	Activates pro-inflammatory responses	[52]
Toxins	Secreted autotransporter toxin (SAT)	Induces vacuolation and disrupts cell morphology	[53]
	Cytolethal distending toxin (CDT)	Damages DNA and stops division, leading to cell distension and death	[54]
	CNF1 (cytotoxic-necrotizing factor 1)	Binds to the BCAM receptor and activates Rho GTPases, further triggering actin-mediated pathogen entry and promoting host cell survival by inhibiting apoptosis	[55,56]
	Hemolysin A (HlyA)	Forms membrane pores to activate host proteases that degrade paxillin and NF-κB, causing cell exfoliation, immune suppression, and caspase-mediated apoptosis	[57,58]
Autotransporters	Antigen Ag43a (a surface-associated adhesin)	Mediates cell-to-cell aggregation through surface-associated binding, promoting the formation of intracellular bacterial biofilms	[59]
	AT UpaG	Binds to bladder uroepithelium and extracellular matrix (fibronectin/laminin), facilitating initial colonization and adherence	[59]
	UpaB, UpaC, and UpaH	Anchors bacteria to the bladder wall, playing a critical role in establishing infection	[59]
Others	TIR domain-containing proteins (Tcp)	Blocks TLR/immune signaling and promotes bacterial survival	[60]
	Outer membrane vesicles (OMVs)	Act as proteoliposome delivery vehicles for toxins and signaling molecules, resulting in improved nutrient acquisition, AMR, and enhanced biofilm formation	[59]

**Table 3:** Formulation composition, regulatory status, and clinical efficacy summaries of leading UPEC vaccine approaches.

Vaccine	Formulation Type	Route of Administration	Current Status	Target Patient Population and Evidence Summary	Reference
OM-89 (Urovaxom)	Formulation contains 6 mg of lyophilized bacterial lysates from 18 separate strains of *E. coli*.	Oral	Currently licensed in over 30 countries worldwide	Demonstrated clinical efficacy in preventing recurrent UTIs, with women comprising the vast majority of the studied trial cohort.	[264,276,277]
Uromune (MV140)	Whole-cell heat-inactivated bacterial suspension containing equal percentages of V121 *Escherichia coli*, V113 *Klebsiella pneumoniae*, V125 *Enterococcus faecalis*, and V127 *Proteus vulgaris* in glycerol, sodium chloride, artificial pineapple flavoring, and water.	Sublingual oral spray	Approved for clinical use in Mexico and the Dominican Republic	Primarily targeted toward female patients experiencing rUTIs, including pre- and postmenopausal individuals, as well as specific patient cohorts with interstitial cystitis/bladder pain syndrome (IC/BPS) or concurrent autoimmune diseases. Three-month daily sublingual course of MV140 significantly reduces UTI episodes and increases UTI-free survival rates.	[278,279]
Urovac (SolcoUrovac)	Cell lysates derived from 10 UPEC strains, including six strains of *E coli*, *K. pneumoniae*. *Proteus mirabilis*. *Proteus morganii*, and *E. faecalis*.	Historically, while identical inactivated bacterial strains were developed for vaginal administration under the name Solco-Urovac, the current StroVac product is licensed and formulated as a deep intramuscular injection	Currently licensed and authorized for clinical use in Germany	Indicated for women experiencing high-frequency recurrent uncomplicated UTIs (more than five episodes per year); it significantly reduces annual recurrence rates to an average of one episode (71.7% success rate). It matches the short-term efficacy of daily Nitrofurantoin prophylactic therapy while providing significantly superior, long-term infection-free survival outcomes at the two-year mark.	[161,280,281,282]
ExPEC10V	10-valent *E. coli* bioconjugate vaccine containing the O-antigen polysaccharides of ExPEC serotypes O1A, O2, O4, O6A, O8, O15, O16, O18A, O25B, and O75.	Intramuscular injection	Evaluated in clinical phase 1/2a development (NCT03819049); currently holds no active commercial license or regulatory approval worldwide	Primarily targeted at older adults (above 60 years) with a history of recurrent UTIs to prevent invasive *E. coli* disease (IED), demonstrating an acceptable safety profile without vaccine-related serious adverse events alongside robust, multi-serotype immunogenicity that peaks by day 15–30 and remains significantly elevated above baseline levels at one year.	[80,158,283]
FimH approaches	Composed of the mannose-binding adhesin protein FimH complexed with the stabilizing chaperone protein FimC, formulated alongside the synthetic adjuvant Phosphorylated HexaAcyl Disaccharide (PHAD).	Subcutaneous	Phase II clinical trial	Case report series evaluated four women with severe, highly recurrent UTIs caused by multidrug-resistant *Enterobacteriaceae*, demonstrating that a multi-dose FimCH vaccine regimen was safe and successfully prevented clinical infection recurrences over a long-term follow-up of up to five years.	[164,283,284]

## Data Availability

No new data were created or analyzed in this study. Data sharing is not applicable to this article.

## Abbreviations

The following abbreviations are used in this manuscript:

UTIs	Urinary tract infections
UPEC	Uropathogenic *Escherichia coli*
AMR	Anti-microbial resistance
HAUTIs	Hospital-acquired urinary tract infections
DNA	Deoxyribonucleic acid
TLR-4	Toll-like receptor-4
NF-kB	Nuclear factor kappa light chain enhancer of activated B cells
dATP	Deoxyadenosine triphosphate
CD14	Cluster of differentiation 14
LPS	Lipopolysaccharide
TIR	Toll/interleukin-1 (IL-1) receptor
MyD88	Myeloid differentiation primary response 88
TIRAP/MAL	TIR domain-containing adaptor protein/MyD88 adaptor-like protein
IRAK	IL-1 receptor-associated kinase
AC-3	Adenylate cyclase-3
ATP	Adenosine triphosphate
cAMP	Cyclic adenosine monophosphate
CREB	cAMP response element-binding protein
PKA	Protein kinase A
PapG	P fimbrial adhesin G
GbO4	Globotetraosylceramide
TRAM	TRIF-related adaptor molecule
MAP kinase	Mitogen-activated protein kinase
IRF-3	Interferon regulatory factor 3
AP-1	Activator protein-1
SAT	Secreted autotransporter toxin
PMNs	Polymorphonuclear neutrophils
rUTIs	Recurrent urinary tract infections
IBCs	Intracellular bacterial communities
QIRs	Quiescent intracellular reservoirs
FAK	Focal adhesion kinase
RAC1	Ras-related C3 botulinum toxin substrate-1
GTP	Guanosine triphosphate
Lcn-2	Lipocalin-2
CNF1	Cytotoxic necrotizing factor 1
BCAM	Basal cell adhesion molecule
CDC42	Cell division cycle 42
HlyA	α-Hemolysin
CDT	Cytolethal distending toxin
AT	Autotransporter
Tcp	TIR domain-containing proteins
OMVs	Outer membrane vesicles
LAMP-1	Lysosome-associated membrane protein-1
TMP-SMX	Trimethoprim–sulfamethoxazole
SXT	Cotrimoxazole
Fol pathway	Folic acid synthesis pathway
CTX-M	Cefotaximases
NDM	New Delhi metallo β-lactamase
ESBL	Extended spectrum β-lactamase
MDR	Multidrug resistant
MurA	UDP-N-acetylglucosamine enolpyruvyl transferase
Cys-115	Cysteine 115
PACs	Proanthocyanidins
ASIR	Age-standardized incidence rate
DALYs	Disability-adjusted life years
ASMR	Age-standardized mortality rate
ASDR	Age-standardized disability rate
